# Dietary pattern in women with polycystic ovary syndrome with and without associated non-alcoholic fatty liver disease: A cross-sectional study

**DOI:** 10.1016/j.clinsp.2023.100288

**Published:** 2023-12-05

**Authors:** Thais Cristine Moura Guimarães, Daniela Oliveira de Lima Taranto, Claudia Alves Couto, Mateus Jorge Nardelli, Ana Lucia Cândido, Cristina de Almeida Hott, Lucilene Rezende Anastácio, Fernando M. Reis, Ana Luiza Lunardi Rocha, Luciana Costa Faria

**Affiliations:** a Pós-Graduação em Ciências Aplicadas à Saúde do Adulto, Universidade Federal de Minas Gerais, Belo Horizonte, MG, Brazil; b Serviço de Diagnóstico por Imagem do Hospital das Clínicas da Universidade Federal de Minas Gerais, Belo Horizonte, MG, Brazil; c Departamento de Clínica Médica, Faculdade de Medicina da Universidade Federal de Minas Gerais, Belo Horizonte, MG, Brazil; d Ambulatório de Doença Hepática Gordurosa Não Alcoólica, Instituto Alfa de Gastroenterologia, Hospital das Clínicas da Universidade Federal de Minas Gerais, Belo Horizonte, MG, Brazil; e Ambulatório de Hiperandrogenismo, Serviço de Endocrinologia, Hospital das Clínicas da Universidade Federal de Minas Gerais, Belo Horizonte, MG, Brazil; f Departamento de Alimentos, Faculdade de Farmácia da Universidade Federal de Minas Gerais, Belo Horizonte, MG, Brazil; g Departamento de Ginecologia e Obstetrícia, Faculdade de Medicina da Universidade Federal de Minas Gerais, Belo Horizonte, MG, Brazil

**Keywords:** Polycystic ovary syndrome, Nonalcoholic fatty liver disease, Diet

## Abstract

**Introduction:**

Women with Polycystic Ovary Syndrome (PCOS) have a higher prevalence of Nonalcoholic Fatty Liver Disease (NAFLD) than the general population. PCOS and NAFLD have common metabolic risk factors, however, the role of diet in NAFLD development is still uncertain in PCOS women.

**Objective:**

To evaluate and compare the dietary patterns and nutritional intake in patients with PCOS with and without NAFLD.

**Method:**

Cross-sectional study that included patients with PCOS diagnosed according to Rotterdam criteria. All participants were submitted to abdominal ultrasound to investigate liver steatosis. Dietary profile was assessed by 24-hour food recall (24hR), and Food Frequency Questionnaire (FFQ). Diet quality was assessed by the Healthy Eating Index (HEI) adapted for the Brazilian population. Physical activity practice was also assessed.

**Results:**

87 participants were included (average age 35.2 ± 5.7 years), among whom, 67 (77%) had NAFLD. The group with PCOS and NAFLD presented higher body mass index (BMI) (34.9 ± 4.5 vs. 30.4 ± 4.9 kg/m2; p = 0.001), Waist Circumference (WC) (103 [97‒113] vs. 95 [87.5‒100] cm; p < 0.001) and were considered physically active less frequently than those without NAFLD (34.3% vs. 60%; p = 0.04). Food intake and dietary patterns assessed by 24hR, FFQ and HEI presented no difference between the groups.

**Conclusions:**

PCOS women with coexistent NAFLD had higher BMI, WC and were less physically active than those without NAFLD. Dietary evaluation showed that PCOS women with NAFLD had no significant difference in macro and micronutrients or food group intake and diet quality in comparison to those without NAFLD.

## Highlights

• The role of diet in NAFLD development is still uncertain in PCOS women.
• Anthropometry and sedentarism were different between PCOS women with and without NAFLD.
• Food intake and dietary patterns were similar between PCOS women with and without NAFLD.

## Introduction

Polycystic Ovary Syndrome (PCOS) is a heterogeneous disorder characterized by hyperandrogenism and ovulatory dysfunction. It is a common condition in women of fertile age, with an estimated prevalence of 6% to 18%.^1^ Multiple genetic, metabolic and hormonal factors interact in PCOS, with frequent association with obesity, Insulin Resistance (IR), type 2 Diabetes Mellitus (DM), glucose intolerance, arterial hypertension and increased cardiovascular risk.^2^ Adipose tissue plays an important role in the development and maintenance of PCOS, since adiposity is associated with symptoms severity in women with PCOS, and even modest reductions in weight generally impact on significant improvements in menstrual regularity, fertility and hyperandrogenic features.^3^

Nonalcoholic Fatty Liver Disease (NAFLD) is a clinical-pathological condition that can manifest as simple steatosis (*i.e.,* hepatic parenchymal lipid accumulation) or as Nonalcoholic Steatohepatitis (NASH), characterized by steatosis, hepatocyte injury (*i.e.,* ballooning), inflammation and fibrosis, with potential progression to liver cirrhosis and hepatocellular carcinoma.^4^ NAFLD is the most common liver disease worldwide, with an estimated prevalence of 25% globally, and 32% in South America.^5^ The disease is strongly associated with obesity, DM, IR and is considered the hepatic manifestation of Metabolic Syndrome (MS).^6^

Many studies have shown a strong association between PCOS and NAFLD.^7^ Obesity and IR are considered major factors related to the concomitance of these two conditions. IR, a cornerstone of MS, is considered the key event, present in 50% to 80% of women with both diagnoses.^8^ In fact, hyperandrogenism, which is the main feature of PCOS, is associated with IR and may be an additional contributing factor to the development of NAFLD in women with PCOS.^2^

The high prevalence of obesity in women with PCOS may reflect inadequate dietary habits, although available data in such populations are still sparse and contradictory.^9^ Most studies found that daily energy and macronutrient intake did not differ between women with and without the syndrome,^10^, ^11^, ^12^ although minor discrepancies, regarding specific nutrients and food categories^10^,^11^ have been observed. Whether women with PCOS have different patterns of dietary intake and food preferences with respect to the unaffected population remains to be established. In this, sense, the aims of this study were to characterize and compare the nutritional intake and dietary patterns of women with PCOS with and without associated NAFLD.

## Methods

This is a cross-sectional study developed in the Hyperandrogenism Clinic of a tertiary University Hospital from September 2016 to November 2018. The study was approved by the Ethics Committee in Research in Humans from the Federal University of Minas Gerais (CAAE: 57599916.0.0000.5149).

### Study population

All patients with a diagnosis of PCOS attending the Hyperandrogenism Clinic during the period of the study development, who filled the inclusion criteria (age 18‒46 years, diagnosis of PCOS established according to the Rotterdam criteria^13^) were invited to participate and included after giving informed consent. The exclusion criteria were alcohol intake higher than 20 g/day or 140 g/week, other chronic liver disease, use of drugs that may cause liver steatosis.

### Clinical, laboratory and anthropometric assessment

Participants' medical history was obtained from medical records and interviews, including diagnosis of hypertension, DM, dyslipidemia, history of alcohol consumption, and laboratory tests. Laboratory tests included Fasting Plasma Glucose (FPG), glycohemoglobin, Triglycerides (TG), Total Cholesterol (TC), High-Density Lipoprotein (HDL) and Low-Density Lipoprotein (LDL) cholesterols.

The diagnosis of MS was defined according to the criteria adopted by the International Diabetes Federation.^14^ The criteria proposed by the American Diabetes Association (ADA) were used to define diabetes.^15^ Impaired Fasting Glucose (IFG) was defined as FPG ≥ 100 mg/dL and < 126 mg/dL. IR was estimated with the Lipid Accumulation Product (LAP), calculated as (Waist Circumference [WC] – 58) × TG (mmoL/L). The occurrence of IR was considered if LAP ≥ 34.5.

Anthropometric evaluation was performed, including the measurement of weight, height and WC, according to the criteria set by the World Health Organization.^16^ Weight was measured on a properly calibrated mechanical scale, with an individual in an upright position, with as few clothes as possible, barefoot, or with light socks. The calibration was performed manually before each weighing, by the researcher. Height was measured in the stadiometer of the same equipment, with the individual standing upright, barefoot or wearing socks. WC was measured using a two-meter anthropometric tape, inelastic for measuring the midpoint between the last rib and the iliac crest, with the patient standing at maximum expiration, with the arms extended along the body. After obtaining these measurements, body mass index [BMI = weight (kg)/height^2^ (m)] was calculated and classified as normal (18.5–24.9 kg/m^2^), overweight (25.0–29.9 kg/m^2^), obesity class I (30.0–34.9 kg/m^2^), obesity class II (35.0–39.9 kg/m^2^) and obesity class III (≥ 40.0 kg/m^2^). Central obesity was defined as WC > 80 cm.

### NAFLD diagnosis

All participants underwent abdominal ultrasonography. The exam was performed by two radiologists with more than 15 years of experience in ultrasonography, who were unaware of the diagnosis of PCOS, using a 3.5 MHz convex probe (Toshiba Xario, Tochigi, Japan). Hepatic steatosis was considered absent when the echogenicity of the hepatic parenchyma was equal to that of the renal cortex. Fatty liver was determined in the presence of a higher echogenicity in the hepatic parenchyma compared with the renal cortex and with impaired visualization of the intrahepatic vessels and diaphragm. All PCOS patients with steatosis were assessed by a hepatologist to confirm NAFLD diagnosis by clinical and laboratory exclusion of other chronic liver diseases,^4^ such as significant alcohol consumption, viral hepatitis (*i.e.,* hepatitis C and B viruses serology), autoimmune liver diseases (*i.e.,* autoantibodies and protein electrophoresis), hemochromatosis (*i.e.,* iron overload), Wilson's disease (*i.e.,* ceruloplasmin) and alpha-1-antitrypsin deficiency.

### Dietary assessment

Participants underwent dietary assessment using the 24-hour food recall (24hR) and the Food Frequency Questionnaire (FFQ).^17^ Data collected were used to compare nutritional intake, food intake, and dietary patterns between PCOS patients with NAFLD and without NAFLD.

The 24hR assesses food and beverages consumed in the last 24 hours, thus reflecting recent consumption. The information was converted from homemade measures to grams. The professional software *Dietbox Nutrition*, recommended by the Brazilian Nutrition Association, was used to evaluate the data collected according to the Brazilian food composition guide.^18^ Nutritional intake calculations included: ingested calories, carbohydrates, proteins, lipids, sugar, fiber, magnesium, calcium, sodium, zinc, phosphorus, manganese, potassium, selenium, vitamin D, vitamin E, vitamin C, vitamin A, vitamin B1, vitamin B12, vitamin B2, vitamin B3, vitamin B6, vitamin B9, polyunsaturated fatty acids, monounsaturated fatty acids, and saturated fat. Total energy intake and total protein intake were also evaluated per kilogram of adjusted body weight (weight at BMI 24.9 kg/m^2^+25% of the excessive weight).^19^

The FFQ used is a semi-quantitative questionnaire validated in the Brazilian population,^17^ which includes a list of more than 80 food items and provides information on long-term nutritional habits. The participants were asked to report how often, on average, they consumed each of the foods and beverages included in the FFQ during the previous year (*i.e.,* daily/weekly/biweekly/rarely/never). Portion sizes were also collected. The nutrient components of each food item were also taken from the Brazilian food composition guide.^18^ Portions of foods consumed in this dietary questionnaire were divided into eight groups, according to the food guide for the Brazilian population:^20^ cereals; vegetable oils; fruits; greens and vegetables; poultry, fish and meat; legumes and oilseeds; meat, fat and butter; refined sugar and salt. Oil consumption was assessed by the volume of liters used per month divided by family members. The consumption of sugar was measured from the durability of each package, calculated in grams, and divided by the number of family members.

Adapted Healthy Eating Index (HEI) was calculated based on data from FFQ and 24hR.^21^ The index assesses 12 components, 8 of which refer to food groups (*i.e.,* cereals, vegetables, fruits, legumes, meat, dairy products, oils and fats, sugars, and sweets), which were extracted from FFQ. Total fat, saturated fat, cholesterol, and a variety of foods in the diet were quantified from data obtained in R24h. Each component was evaluated by a score that varied from zero to 10. If the food group consumption was equal to or greater than the recommended, the individual received 10 points for the evaluated category. If there was no consumption of the food group, the received score was zero. Intermediate values were calculated proportionally to the recommended amount of consumption. In the case of total and saturated fat and cholesterol, sugars and sweets, the score was lower the greater the consumption. HEI score classifies dietary habits as good quality (greater than 100 points), improvement required (71‒100 points), and poor quality (less than 71 points).

### Physical activity assessment

Participants were asked about the weekly frequency of aerobic physical activities and were considered physically active if they practiced 150 min or more/week of moderate-intensity aerobic exercises during at least three weekly sessions.

### Statistical analysis

Statistical analysis was performed with SPSS for Windows, version 18.0 (SPSS Inc., Chicago, IL, USA). Shapiro-Wilk test was used to test the normality of data. Continuous variables were presented as means and Standard Deviation (SD) or medians and ranges. Categorical variables were presented as absolute numbers and percentages. The Levine test was used to evaluate homogeneity of variance. Univariate analysis was performed to assess the association between total calories, macronutrients and micronutrient intake and the presence of liver steatosis. Group differences (between patients with and without NAFLD) for continuous variables were analyzed using a *t*-test or the Mann-Whitney *U* test and, for categorical variables, using the X^2^-test or Fisher's exact test, as appropriate.

## Results

Initially, 91 individuals diagnosed with PCOS were selected. Among these, one was excluded due to a previous diagnosis of hepatitis B and three were excluded due to non-attendance for abdominal US. The final sample enrolled 87 participants.

From the total, 67 (77.0%) presented liver steatosis in the abdominal US. Metabolic comorbidities were as follows: diabetes mellitus in 11 (12.6%), dyslipidemia in 14 (16.1%), arterial hypertension in 22 (25.3%) and MS in 43 (49.4%). Obesity by BMI was observed in 70 (80.5%) participants, and, central obesity, in 79 (90.8%). Thirty-five (40.2%) participants were considered physically active.

Participants with hepatic steatosis (77.0%), when compared to those without, presented higher values of BMI (34.9 ± 4.5 vs. 30.4 ± 4.9 kg/m^2^, p < 0.001), WC (103 [97‒113] vs. 95 [87‒100] cm, p < 0.001), serum triglycerides (140 [113‒193] vs. 92 [64‒151] mg/dL, p = 0.003) and total cholesterol (196 ± 34 vs. 176 ± 27 mg/dL, p = 0.021), besides higher frequencies of obesity grade 2 and 3 (37.3% vs. 15.0%, 13.4% vs. 0.0%, respectively, p = 0.003), central obesity (95.5% vs. 75.0%, p = 0.014), MS (59.2% vs. 22.1%, p = 0.003), IR by LAP (93.8% vs. 61.1%, p = 0.001) and sedentarism (65.7% vs. 40.0%, p = 0.040).

Clinical, anthropometric, and laboratory data of the PCOS participants and the comparison between presence and absence of NAFLD diagnosis are presented in Table 1.

**Table 1:** Clinical, anthropometric and laboratory data of participants with Polycystic Ovary Syndrome (PCOS), PCOS and Non-Alcoholic Fatty Liver Disease (NAFLD) and PCOS without NAFLD.

	All participants with PCOS (*n* = 87)	PCOS and NAFLD (*n* = 67)	PCOS without NAFLD (*n* = 20)	*p*-value
**Age (years)**	35.2 ± 5.7	35.8 ± 5.5	33.3 ± 6.3	0.084^a^
**BMI (kg/m^2^)**	33.8 ± 4.9	34.9 ± 4.5	30.4 ± 4.9	**< 0.001**^a^
Eutrophic	3 (3.4)	0 (0.0)	3 (15.0)	**0.003**^d^
Overweight	14 (16.1)	11 (16.4)	3 (15.0)	
Obesity grade 1	33 (37.9)	22 (32.8)	11 (55.0)	
Obesity grade 2	28 (32.2)	25 (37.3)	3 (15.0)	
Obesity grade 3	9 (10.3)	9 (13.4)	0 (0.0)	
**Waist circumference (cm)**	102.0 (95.0‒112.0)	103.0 (97.0‒113.0)	95.0 (87.5‒100.0)	**< 0.001**^b^
Central obesity	79 (90.8)	64 (95.5)	15 (75.0)	**0.014**^d^
**Physically active**	35 (40.2)	23 (34.3)	12 (60.0)	**0.040**^c^
**Hyperandrogenism**	67 (77.0)	53 (79.1)	14 (70.0)	0.383^d^
**Metabolic syndrome**	43 (50.6)	39 (59.1)	4 (21.1)	**0.003**^c^
**Insulin resistance**	72/83 (86.7)	61/65 (93.8)	11/18 (61.1)	**0.001**^d^
**Diabetes mellitus**	11 (12.6)	10 (14.9)	1 (5.0)	0.241^d^
**Arterial hypertension**	22 (25.3)	18 (26.9)	4 (20.0)	0.535^c^
**Dyslipidemia**	14 (16.1)	12 (17.9)	2 (10.0)	0.507^d^
**Triglycerides (mg/dL)**	133 (101‒180)	140 (113‒193)	92 (64‒151)	**0.003**^b^
**Total cholesterol (mg/dL)**	191 ± 33	196 ± 34	176 ± 27	**0.021**^a^
**LDL-cholesterol (mg/dL)**	116 ± 29	118 ± 29	108 ± 29	0.177^a^
**HDL-cholesterol (mg/dL)**	45 ± 9	45 ± 9	47 ± 7	0.506^a^
**Fasting glycemia (mg/dL)**	89 (83‒95)	89 (84‒98)	85 (82‒93)	0.060^b^
**Glycohemoglobin (%)**	5.5 ± 0.4	5.6 ± 0.4	5.4 ± 0.3	0.096^a^

Data are presented as absolute number/available data (percentage), mean ± standard deviation or median (interquartile range). Insulin resistance was defined by LAP ≥ 34.5; BMI, Body Mass Index; HDL, High-Density Lipoprotein; LDL, Low-Density Lipoprotein; NAFLD, Nonalcoholic Fatty Liver Disease; PCOS, Polycystic Ovary Syndrome; WC, Waist Circumference.

a Student t-test.

b Mann-Whitney test.

c Chi-Square test.

d Fisher's exact test.

### Food intake

Results of nutritional intake evaluated by 24hR in PCOS participants with and without NAFLD are demonstrated in Table 2. There were no differences in micro and macronutrient intake between the two groups.

**Table 2:** Dietary intake by 24-hour food recall in participants with Polycystic Ovary Syndrome (PCOS), with and without associated Nonalcoholic Fatty Liver Disease (NAFLD).

	PCOS (*n* = 87)	PCOS and NAFLD (*n* = 67)	PCOS without NAFLD (*n* = 20)	*p*-value
**Energy (kcal)**	1445 (1082‒1817)	1415 (1013‒1821)	1494 (1259‒1815)	0.432^c^
**Energy (kcal/kg)**	21.4 ± 9.3	21.1 ± 9.4	22.6 ± 9.0	0.543^b^
**Protein (g)**	62 (47‒83)	59 (44‒83)	76 (57‒93)	0.067^c^
**Protein (g/kg)**^a^	0.96 ± 0.46	0.9 ± 0.4	1.1 ± 0.5	0.122^b^
**Carbohydrates (g)**	193 ± 83	189 ± 86	207 ± 70	0.402^b^
**Lipids (g)**	45 (34‒64)	46 (34‒65)	45 (34‒64)	0.780^c^
**Cholesterol (g)**	136 (97‒247)	128 (95‒293)	147 (102‒196)	0.905^c^
**Monounsaturated fat**	13 (10‒21)	14 (10‒21)	13 (6‒15)	0.126^c^
**Saturated fat (g)**	15 (10‒21)	15 (10‒21)	14 (10‒23)	0.495^c^
**Polyunsaturated fatty acid (g)**	8 (5‒14)	8 (5‒14)	7 (6‒11)	0.704^c^
**Sugar (g)**	29 (15‒54)	30 (13‒59)	28 (18‒40)	0.996^c^
**Fiber (g)**	14 (10‒19)	13 (9‒19)	15 (12‒21)	0.252^c^
**Calcium (mg)**	401 (241‒631)	404 (269‒624)	401 (176‒835)	0.862^c^
**Magnesium (mg)**	189 ± 85	181 ± 86	218 ± 76	0.091^b^
**Manganese (mg)**	1.4 (1.1‒2.4)	1.6 (1.1‒2.5)	1.3 (0.9‒1.9)	0.386^c^
**Phosphorus (mg)**	708 (488‒1001)	697 (490‒996)	746 (484‒1235)	0.558^c^
**Potassium (mg)**	1805 (1256‒2390)	1776 (1233‒2387)	1938 (1482‒2520)	0.509^c^
**Selenium (µg)**	66 (48‒106)	64 (48‒99)	74 (42‒116)	0.468^c^
**Sodium (mg)**	1456 (1052‒2157)	1338 (1066‒1883)	2022 (919‒2776)	0.201^c^
**Zinc (mg)**	7 (5‒11)	7 (5‒11)	7 (3‒10)	0.515^c^
**Vitamin A (µg)**	253 (142‒538)	243 (148‒533)	302 (123‒580)	0.895^c^
**Vitamin B1 (mg)**	1.0 (0.7‒1.4)	1.0 (0.7‒1.4)	1.0 (0.7‒1.4)	0.841^c^
**Vitamin B2 (mg)**	1.0 (0.7‒1.4)	1.0 (0.7‒1.5)	1.0 (0.7‒1.4)	0.704^c^
**Vitamin B3 (mg)**	14.4 (8.8‒18.8)	14 (8‒20)	14 (9‒16)	0.481^c^
**Vitamin B6 (mg)**	1.1 (0.8‒1.5)	1.1 (0.8‒1.5)	1.1 (0.7‒1.8)	0.508^c^
**Vitamin B9 (mg)**	180 (112‒255)	188 (108‒258)	160 (115‒217)	0.588^c^
**Vitamin B12 (µg)**	2.0 (1.1‒4.0)	2.0 (1.0‒3.9)	2.1 (1.6‒4.1)	0.438^c^
**Vitamin C (µg)**	42 (22‒100)	49 (19‒105)	37 (23‒86)	0.920^c^
**Vitamin D (µg)**	1.3 (0.6‒2.3)	1.3 (0.6‒2.4)	1.2 (0.5‒2.2)	0.602^c^
**Vitamin E (µg)**	6 (4‒10)	6 (3‒11)	6 (5‒8)	0.920^c^

Data are presented as mean ± standard deviation or median (interquartile range).

NAFLD, Nonalcoholic Fatty Liver Disease; PCOS, Polycystic Ovary Syndrome.

a Per quilogram of adjusted body weight.

b Student t-test.

c Mann-Whitney test.

The food group consumption calculated from the FFQ results are shown in Table 3. There was no significant difference between the groups of patients with and without NAFLD.

**Table 3:** Dietary intake by food frequency questionnaire in participants with Polycystic Ovary Syndrome (PCOS), with and without associated Nonalcoholic Fatty Liver Disease (NAFLD).

	PCOS (*n* = 87)	PCOS and NAFLD (*n* = 67)	PCOS without NAFLD (*n* = 20)	*p*-value
**Cereals**	4 (0‒15)	4 (0‒8)	4 (1‒15)	0.975^a^
**Vegetable oils**	22 (0‒90)	30 (1‒90)	15 (0‒30)	0.405^a^
**Fruit**	2.1 ± 1.4	2.1 ± 1.4	2,0 ± 1.6	0.823^b^
**Vegetables greens**	4 (0‒13)	4 (0‒13)	4 (0‒13)	0.388^a^
**Poultry, fish, meats**	1 (0‒15)	1 (0‒15)	1 (1‒3)	0.704^a^
**Oil seeds**	1 (0‒7)	1 (0‒7)	1 (0‒2)	0.293^a^
**Meat fats**	1 (0‒3)	0.7 (0‒2)	1 (0‒3)	0.129^a^
**Refined sugar and salt**	1 (0‒3)	1 (0‒3)	1 (0‒2)	0.113^a^

Data are expressed as absolute numbers and refer to portions.

a Mann-Whitney test.

b Student t-test.

The results of Adapted Healthy Eating Index for the Brazilian population by diet components are shown in Table 4. In total fat intake, diet quality was higher in patients without NAFLD (10 points vs. 5 points, p = 0.066), but the difference was not statistically significant. In the classification of diet quality (Table 5), there was no difference between the groups of patients with and without NAFLD.

**Table 4:** Evaluation of Polycystic Ovary Syndrome (PCOS) patients’ dietary intake according to the healthy eating index score for each food category.

	PCOS with NAFLD (*n* = 67)	PCOS without NAFLD (*n* = 20)	*p*-value
**Cereal, bread, tubercules, and roots (points)**	1.90 (1.43‒10.0)	1.89 (1.50‒2.27)	0.700
**Vegetables (points)**	9.51 (5.36‒10.0)	10.0 (5.84‒10.0)	0.416
**Fruits (points)**	6.67 (3.33‒10.0)	6.54 (3.39‒10.0)	0.958
**Meat (points)**	10.0 (10.0‒10.0)	10.0 (10.0‒10.0)	0.464
**Dairy and derivates (points)**	3.3 (0.0‒6.7)	3.3 (0.0‒6.7)	0.445
**Sugar and sweets (points)**	10.0 (10.0‒10.0)	10.0 (5.7‒10.0)	0.341
**Oil and fat (points)**	0.0 (0.0‒10.0)	10.0 (0.0‒10.0)	0.231
**Total fat (points)**	5.0 (5.0‒10.0)	10.0 (5.0‒10.0)	0.066
**Saturated fat (points)**	10.0 (5.0‒10.0)	10.0 (5.0‒10.0)	0.300
**Cholesterol (points)**	10.0 (5.0‒10.0)	10.0 (10.0‒10.0)	0.060
**Diet variety (points)**	11.0 (9.0‒14.0)	12.5 (9.7‒15.2)	0.277

NAFLD, Nonalcoholic Fatty Liver Disease.

Mann-Whitney test was performed.

**Table 5:** Classification of diet quality of the PCOS and PCOS groups with associated NAFLD by the Adapted Healthy Eating Index for the Brazilian population.

Classification of diet quality	PCOS (*n* = 87)	PCOS with NAFLD (*n* = 67)	PCOS without NAFLD (*n* = 19)	*p*-value
Bad quality, n (%)	14 (16.0)	13 (19.4)	1 (5.2)	0.158
Needs improvement, n (%)	62 (71.2)	45 (67.2)	17 (89.5)	
Good quality, n (%)	10 (11.5)	9 (13.4)	1 (5.2)	

NAFLD, Nonalcoholic Fatty Liver Disease; PCOS, Polycystic Ovary Syndrome.

Chi-Square test was performed.

## Discussion

This study showed that PCOS patients who also have NAFLD, when compared to those without NAFLD, are more likely to present higher BMI, WC, serum triglycerides, and total cholesterol, besides higher frequencies of obesity and MS. There was no difference in dietary patterns and food/nutrient intake between both groups when assessed by 24h-FR nor FFQ, or when comparing them based on dietary quality or adequacy indexes. However, patients with NAFLD were considered less physically active than those without NAFLD.

NAFLD was diagnosed in 77% of the study participants, as already published by the present group.^22^ Other studies around the world showed fatty liver frequencies, among women with PCOS, of 40%‒50%.^23^, ^24^, ^25^ In the Brazilian population, a previous investigation showed that fatty liver was observed in 24% of patients with PCOS.^26^

NAFLD is considered the hepatic manifestation of MS, therefore low-quality lifestyle is common in individuals with fatty liver and may play a role in its pathophysiology. Reduction of total energy intake, reduction of total fat, trans fatty acids, saturated fatty acids and fructose are recommended as a form of treatment.^4^ However, the specific macronutrient composition of diet appears to be less relevant than sustained weight loss, and it is suggested that a reduction of at least 5% of body weight is required to improve steatosis, 7% to regression of histopathological features of NASH and more than 10% to fibrosis improvement.^8^

The role of diet in the development of PCOS is still uncertain. It has been previously observed that women with PCOS do not have higher caloric intake than women without PCOS.^10^ However, in other studies, caloric surplus has been associated with PCOS diagnosis, when compared to controls.^27^,^28^ This investigation showed that PCOS women with NAFLD did not consume more calories than those without NAFLD, based on 24hR. Although the development of NAFLD has been associated with a caloric surplus,^4^ in the present analysis caloric intake was not associated with NAFLD diagnosis in patients with PCOS, when compared to those without NAFLD.

Previous studies that analyzed macronutrient intake have shown that PCOS women have a lower intake of fibers when compared to controls. ^29^,^30^ Fiber consumption, in addition to helping to improve the glycemic response, may favor weight control, through an increase in postprandial satiety and consequent reduction in total caloric consumption, a desirable factor in the treatment of NAFLD.^31^ The present investigation showed that PCOS women with NAFLD did not present a different intake of fibers when compared to those without NAFLD, by any dietary assessment method.

Previous investigation has been performed for micronutrient intake and PCOS subjects, when compared to controls, presented lower vitamin D, B9 and sodium consumption.^29^,^32^ In the present study, no micronutrient intake difference was observed among PCOS subjects with and without NAFLD. Higher vitamin C intake has been associated with lower frequencies of NAFLD in the general population, and even with lower prevalence of steatohepatitis.^33^,^34^ A Randomized clinical trial with vitamin C and vitamin E treatment in patients with steatohepatitis has shown an improvement in NAFLD progression in six months,^35^ the study does not make it possible to conclude that vitamin C plays a role in preventing steatohepatitis progression, because it was combined with vitamin E, which is a recommended treatment for steatohepatitis.^4^

In this study, none of the dietary patterns investigated (energy, nutrients, food groups nor HEI) were different among PCOS subjects with and without NAFLD. Dietary assessment tools used in this study – 24hR and FFQ – are commonly used to capture habitual dietary intakes, however, they have limitations related to systematic error analyses, such as memory bias and misreporting. Also, a single 24hR is not representative of a typical diet^36^ and the combination of dietary methods to reduce bias is advised. The strength of observed associations between diet and health outcomes explored using dietary methods alone may be biased toward the null.^37^ The bias of dietary assessment can increase the chance of false negative results,^38^ thereby necessitating very large cohort studies to assess relationships between diet and disease.

In the present study, participants without NAFLD were more frequently physically active than those with NAFLD. Many studies have demonstrated that regular exercises such as aerobic activities, resistance exercise, and flexibility training improve NAFLD independent of weight loss.^39^ The molecular mechanisms by which exercise affects the liver remain unclear. Reduced intrahepatic fat content, decreased level of NAFLD-induced oxidative stress, inflammation and apoptosis are the main possible mechanisms of exercise effect on NAFLD treatment.^40^

It has been demonstrated that PCOS patients present more precarious nutritional education than controls,^41^ and that dietary and lifestyle interventions for PCOS women can improve their hormonal profile, besides reducing BMI, fasting glucose, insulin resistance and even improve anovulatory symptoms and hirsutism.^42^ Moreover, caloric restriction, independent of weight loss, has been shown to improve reproductive disorders, dyslipidemia, and insulin resistance in PCOS women.^43^ The present findings showed that differences between caloric, macro and micronutrient intake among PCOS patients with NAFLD were not markedly significant when compared to those without NAFLD. However, further studies are required to investigate if the impact of dietary and lifestyle interventions can be different between these two groups, in order to determine the influence of NAFLD coexistence in the management of PCOS.

The present study was limited by the small sample size. Another limitation was the dietary assessment methods. Recalls and FFQ present an inherent difficulty since patients tend to underestimate the number and the amount of ingested portions. Also, the method to evaluate the practice of physical activity was a direct question about the time and frequency of aerobic physical activities performed during the week. Resistance training was not taken into account. Lastly, the authors utilized abdominal ultrasonography for NAFLD diagnosis, which is known to be a useful method in clinical practice, however, it only presents a reasonable sensitivity to detect steatosis when it is present in more than 30% of the liver parenchyma, so it may have underestimated NAFLD prevalence among PCOS patients and, furthermore, the accuracy of ultrasonography for diagnosis of liver steatosis is reduced in patients with obesity, which represents the profile of the patients in the present study.^44^ The authors have not reported liver steatosis ultrasonographic graduation because it lacks clinical relevance and is also not very accurate.^45^

In conclusion, PCOS women with NAFLD, when compared to those without NAFLD, were less physically active and had no difference in dietary evaluation when assessed by 24h-FR nor FFQ, or when comparing them based on dietary quality or adequacy indexes.

## Authors' contributions

Conceptualization: TCMG, CAC, ALC, FMR, ALLR, LCF. Data curation: TCMG, DOLT, ALC, CAH. Formal analysis: TCMG, MJN, LRA, LCF. Investigation: TCMG, DOLT, CAC, MJN, ALC, CAH, LRA, FMR, ALLR, LCF. Methodology: TCMG, DOLT, CAC, ALC, LRA, FMR, ALLR, LCF. Project administration: TCMG, CAC, ALC, FMR, ALLR, LCF. Supervision: CAC, ALC, CAH, FMR, ALLR, LCF. Writing – original draft: TCMG, MJN, LCF. Writing – review & editing: TCMG, MJN, LRA, ALLR, LCF.

## Ethical approval

This study was approved by the Ethics Committee in Research in Humans from Federal Universidade de Minas Gerais (CAAE: 57599916.0.0000.5149).

## Study guidelines

This study followed the STROBE statement for cross-sectional studies.

## Conflicts of interest

The authors declare no conflicts of interest.
